# Ubiquitous Emergency Medical Service System Based on Wireless Biosensors, Traffic Information, and Wireless Communication Technologies: Development and Evaluation

**DOI:** 10.3390/s17010202

**Published:** 2017-01-21

**Authors:** Tan-Hsu Tan, Munkhjargal Gochoo, Yung-Fu Chen, Jin-Jia Hu, John Y. Chiang, Ching-Su Chang, Ming-Huei Lee, Yung-Nian Hsu, Jiin-Chyr Hsu

**Affiliations:** 1 Department of Electrical Engineering, National Taipei University of Technology, Taipei 10608, Taiwan; thtan@ntut.edu.tw (T.-H.T.); darren.jj.hu@mail.foxconn.com (J.-J.H.); 2 Department of Dental Technology and Materials Science, Central Taiwan University of Science and Technology, Taichung City 40601, Taiwan; yfchen@ctust.edu.tw; 3 Department of Computer Science and Engineering, National Sun Yat-Sen University, Kaohsiung 80424, Taiwan; chiang@cse.nsysu.edu.tw; 4 Department of Data & Broadband Maintenance Center, Chunghwa Telecom Hsinchu Business Group, Hsinchu City 300, Taiwan; csc8100@cht.com.tw; 5 Department of Urology, Feng Yuan Hospital, Ministry of Health and Welfare, Taichung 42055, Taiwan; dire@fyh.mohw.gov.tw; 6 Department of Family Medicine, Taichung Hospital, Ministry of Health and Welfare, Taichung 403, Taiwan; hbtcf00300@taichung.gov.tw; 7 Department of Internal Medicine, Taipei Hospital, Ministry of Health and Welfare, New Taipei City 242-13, Taiwan; dire@tph.mohw.gov.tw; 8 Department of Health Services Administration, China Medical University, Taichung 40402, Taiwan

**Keywords:** ubiquitous emergency medical service, Google Maps, 3.5 G, WiMax, traffic guiding subsystem, speech recognition

## Abstract

This study presents a new ubiquitous emergency medical service system (UEMS) that consists of a ubiquitous tele-diagnosis interface and a traffic guiding subsystem. The UEMS addresses unresolved issues of emergency medical services by managing the sensor wires for eliminating inconvenience for both patients and paramedics in an ambulance, providing ubiquitous accessibility of patients’ biosignals in remote areas where the ambulance cannot arrive directly, and offering availability of real-time traffic information which can make the ambulance reach the destination within the shortest time. In the proposed system, patient’s biosignals and real-time video, acquired by wireless biosensors and a webcam, can be simultaneously transmitted to an emergency room for pre-hospital treatment via WiMax/3.5 G networks. Performances of WiMax and 3.5 G, in terms of initialization time, data rate, and average end-to-end delay are evaluated and compared. A driver can choose the route of the shortest time among the suggested routes by Google Maps after inspecting the current traffic conditions based on real-time CCTV camera streams and traffic information. The destination address can be inputted vocally for easiness and safety in driving. A series of field test results validates the feasibility of the proposed system for application in real-life scenarios.

## 1. Introduction

With the increasing amount of accidents, the quality of emergency medical service (EMS) plays a crucial role in saving lives. The EMS response time is defined as the period between the time when a call is received by an EMS provider and the arrival time of an ambulance to an emergency site [1,2,3]. Reducing the response time by just one minute increases the survival rate of the patients with sudden cardiac arrest by 24% [4]. Several studies concluded that the response time is the most important factor for reducing mortality and morbidity rates [5,6,7]. The second important factor is to provide an adequate pre-hospital treatment for the patients by paramedics in an ambulance as well as a medical equipment preparation for a particular treatment at a hospital.

In the past decades, various mobile telemedicine systems [8,9,10,11,12,13,14,15,16,17] have been proposed to improve the quality of EMS using information and communication technology (ICT). Kyriacou et al. [8] proposed a multi-purpose healthcare telemedicine system. Even though a large number of biosignals and still images of the patient are transmitted through the GSM mobile network, the average transmission time for an image, ranging from 18 to 26 s through the GSM link, is unacceptable for a timely emergency medical service. Chu et al. [9] developed a tele-trauma system that provided pre-hospital trauma care by transmitting the patient’s real-time video, medical images and electrocardiogram (ECG) over a 3 G wireless communication network; however, an average data rate during rush hours were as low as 50–60 Kbits/s. Mandellos et al. [10] proposed a mobile telemedicine system for a pre-hospital treatment via GSM network; however, the interruption rate of connection was high. Thelen et al. [11] implemented an integrated telemedicine system for real-time patient monitoring by employing commercially available off-the-shelf devices and software. The signal delays of ECG and SaO_2_ waveforms were measured via simulated 2 G/3 G networks, and a median of 4.9 s end-to-end signal delay was reported, which exceeded the maximum acceptable delay of 4 s suggested by [12]. Su et al. [13] proposed a tele-ultrasound diagnosis in EMS via 3 G network. Kang et al. [14] implemented a ubiquitous biotelemetry system that integrated biotelemetry devices and 3.5 G (HSDPA) technology for emergency care. Niyato et al. [15] investigated the application of IEEE 802.16/WiMax-based wireless communication technology in telemedicine services. A bandwidth allocation and admission control algorithm for WiMax networks designed specifically for telemedicine services has also ever been presented. Alesanco et al. [12] studied the issue of wireless ECG transmission for a real-time cardiac tele-monitoring by simulated wireless network. Unfortunately, the performance test results of [12,13,14,15] were obtained by simulation. Without field tests, simulation alone cannot reflect the truthful real-world scenario.

Walter et al. [16] developed a mobile stroke unit (MSU) for pre-hospital diagnosis and treatment that consists of a paramedic, a physician trained in stroke medicine, a neuroradiologist, a computed tomography scanner, a complete point-of-care stroke laboratory system, and telemedicine devices using UMTS. Trial-experimental results showed the MSU reduced the time from call to prehospital treatment decision by 41 min (54%). Felzen et al. [17] implemented a telemedicine system in seven ambulances which provided the transmission of vital signs, 12-lead ECG signal, picture, and video stream via second and third generation mobile networks. Overall 539 cases were supported with this system during a five-month study period, and the results verified the feasibility of the system. However, the studies [8,9,10,11,12,13,14,15,16,17] did not use any wireless biosensors and a traffic guiding system. The wireless biosensors can enable a remote accessibility and ease the wiring issues in the ambulance. The traffic guiding system can decrease the response and delivery time.

Recently, Tokuda et al. [18] proposed a smart traffic guiding system for an ambulance. The system broadcasts warning signals to make other vehicles yield their way, transmitting a signal to a traffic control center to change traffic signals to green to reduce the time required to cross intersections and to make such crossings safer, and eventually transmitting vital signals and videos of the patient to a hospital for pre-hospital care. And experimental results revealed the ambulances reached the destination in about a half time under normal traffic conditions. However, implementation of this system at a large scale could be difficult and costly. Similarly, Chen et al. [19] proposed a smart traffic control system for EMS where every car needed a GPS unit. Lim et al. [20] proposed a dynamic ambulance relocation model by rerouting the nearest ambulance to the accident site. Buchenscheit et al. [21] proposed a warning system for an ambulance to cross the traffic light safely. However, feasibility of [18,19,20,21] were tested in a simulation environment.

Although, the aforementioned systems provide extensive services, the following difficulties remain unresolved.

1. Management of wires in the ambulance: in the delivery of first-aid to patients, sensor wires may cause inconvenience for patients and healthcare providers.
2. Ubiquitous accessibility of the patient’s biosignals in remote areas where the ambulance cannot arrive: this would delay the first-aid.
3. Availability of real-time traffic information: Without real-time traffic information, the ambulance cannot reach the accident site in the shortest time.

To address the above difficulties, we propose a new ubiquitous emergency medical service system (UEMS), which is an extended work of our previous preliminary study [22]. The UEMS is an integration of a ubiquitous tele-diagnosis interface and a traffic guiding subsystem. To the best of our knowledge, we have not found a similar system in the literature thus far. In the previous study, a prototype system of the UEMS has been developed by utilizing sensor devices, a smartphone, a webcam, Zigbee, and 3.5 G wireless networks. In this extended work, a new UEMS system is presented based on wireless biosensors, a webcam, a tablet, GPS, Google Maps (GMs), Google speech recognition, WiMax/3.5 G network technologies, and real-time traffic information.

## 2. Materials and Methods

**Figure 1:**
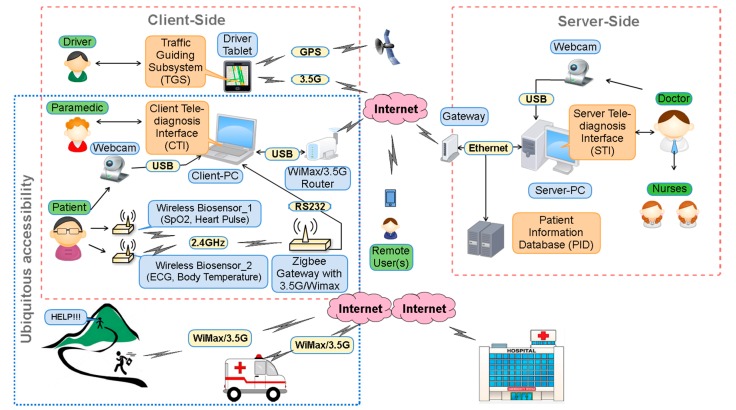
Framework of the proposed system.

Figure 1 shows a framework of the proposed system, which adopts client-server architecture where the client-side, located in an ambulance, consists of wireless biosensors, a webcam, an application server and a tablet, whereas the server-side, installed in a hospital, consists of a remote server and patient information database.

### 2.1. Wireless Sensors and Remote Accessibility

We used wireless biosensors to measure patient’s biosignals. Wireless Biosensor_1 employs an infrared sensor, and measures blood SpO_2_ and heart pulse from the tip of the patient’s finger. Wireless Biosensor_2 measures ECG and body temperature using two electrodes (one-lead ECG measurement) and a temperature sensor around the patient’s waist.

Wireless biosensors and the Zigbee gateway employ a rechargeable lithium ion battery as a power supply and XBee 2 mW Zigbee module with a wire antenna for wireless connection. Output power was adjusted to 3 dBm which would provide 120 m in open space according to the factory specification. Serial baud rate between the biosensors and the gateway was adjusted to 115.2 Kbits/s.

The Zigbee gateway employs an RS232 interface, and a WiMax/3.5 G router as well. The Zigbee gateway transfers data received from the wireless biosensors to the Client-PC via RS232 interface. The 3.5 G/WiMax router can be used in case of remote areas where ambulances cannot directly arrive at, thus paramedics can carry the wireless biosensors and the Zigbee gateway to maintain the wireless connection with the hospital via 3.5 G/WiMax. Thus, pre-hospital treatment can be started in the hospital. This feature provides a ubiquitous accessibility in both general accident sites and remote areas.

### 2.2. Client Tele-Diagnosis Interface

**Figure 2:**
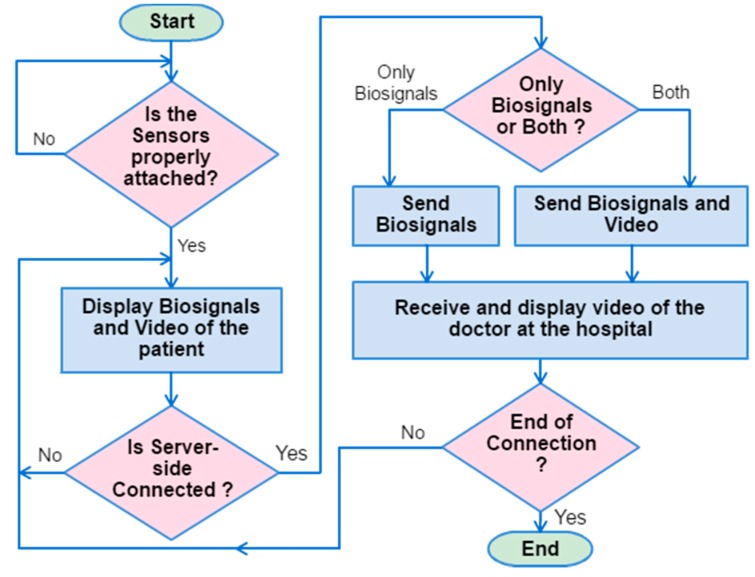
Flowchart of operation for the client tele-diagnosis interface.

**Figure 3:**
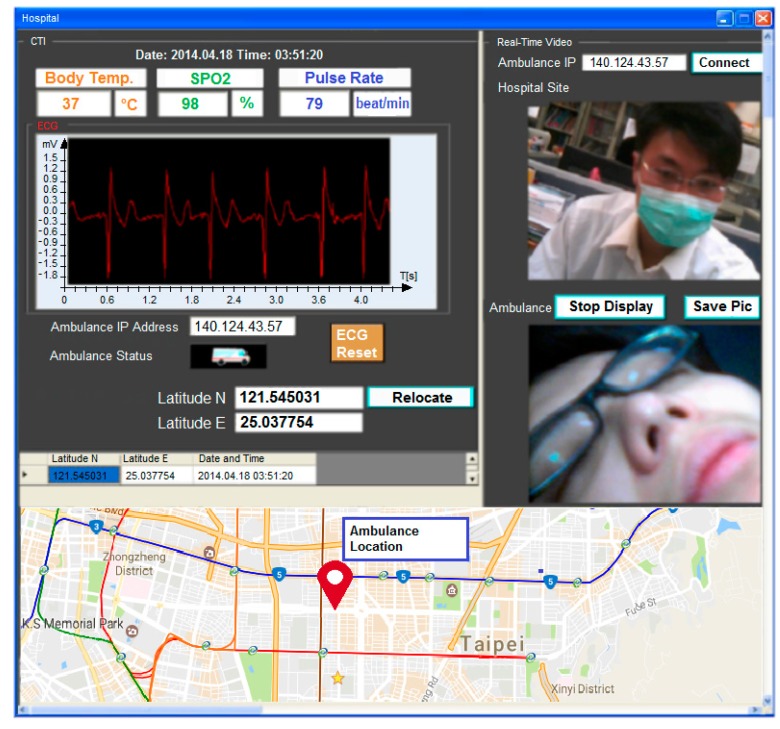
A snapshot of the client tele-diagnosis interface.

A client tele-diagnosis interface (CTI) installed on a portable client PC displays the patient’s biosignals and a real-time video of a patient and a doctor captured by webcams installed in the ambulance and the hospital. Notably, the webcam can be easily moved/zoomed in on an appropriate place in the ambulance. Figure 2 illustrates the flowchart of operation of the CTI. The operation starts by checking all the wireless biosensors being properly attached to the patient’s body. Once the biosignals have been successfully acquired, the CTI will display the ECG signal (see Figure 3), body temperature, oxygen concentration, and heart rate measured by the wireless sensors. All biosignals are delivered in packets which can be distinguished by packet headers and encoding methods. Various webcam video encoding methods can be selected to fit the channel condition. The CTI transmits the real-time video and biosignals simultaneously if the network bandwidth is capable of the transmission; otherwise, only the biosignals will be sent. The CTI also displays the current date and time, the hospital server IP address, and the real-time video of a doctor acquired from the hospital for the instruction of the pre-hospital treatments.

### 2.3. Server Tele-Diagnosis Interface

A server tele-diagnosis interface (STI) installed on a server PC establishes a real-time communication between the hospital and CTI. The STI, which shows the same monitoring signals and videos as illustrated in Figure 3, allows the healthcare providers to communicate with the paramedics in the ambulance for the diagnosis of the patient’s current health status by observing real-time biosignals and videos. First, the STI checks if the connection between the hospital and the CTI is ready. As soon as the connection is ready, the ambulance initiates the connection after receiving the request from the STI. The STI starts to receive packets of the biosignals and real-time videos of the patient from the ambulance via the Winsock [23]. However, in case of insufficient data rate for transmitting both biosignals and videos, the hospital side will choose to only request the biosignals. Videos can be recorded and uploaded to the patient information database at a rate of 30 frames/s and saved as an Audio Video Interleave (AVI) format as a reference, and for further diagnosis, healthcare providers can access and view uploaded video with a private account and password.

### 2.4. Traffic Guiding Subsystem

**Figure 4:**
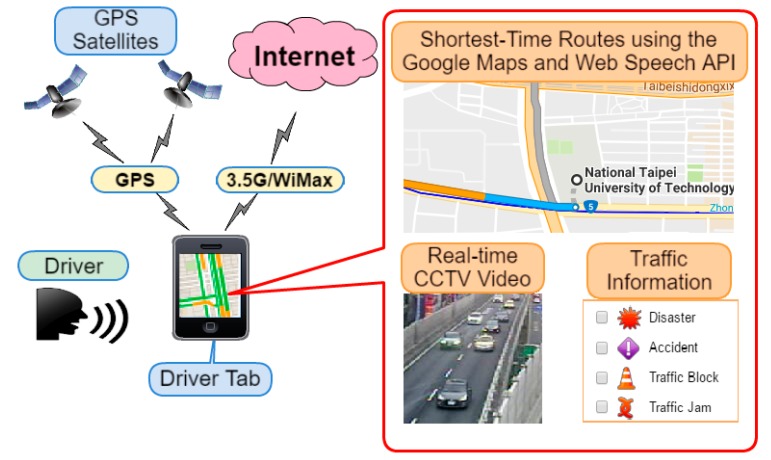
Traffic guiding subsystem. The driver tab is embedded with the GPS and a 3.5 G modem and a microphone.

A traffic guiding subsystem (TGS) installed on the driver tablet embedded with GPS, 3.5 G/WiMax modem, touch screen, and microphone, uses GMs for suggesting the shortest-time routes between current and destination points to the driver, as well as a web speech API (WSA) [24] for providing a faster and easier method to input the destination address in a moving ambulance. The subsystem displays the current location of the ambulance using an embedded GPS, the shortest-time routes, real-time CCTV camera stream and road information provided by E-Traffic Center of Taipei City (see Figure 4). The CCTV camera stream can be acquired by sending requests to the website of the E-Traffic Center. The CCTV camera stream is provided in MJPG-Streamer (JPEG frames) format with 352 × 240 resolution at 10, 12 or 15 frames/s. Moreover, traffic video can be received as a JPEG image upon request.

The shortest-time routes are calculated by GMs using Google Traffic feature [25] on GMs. Google traffic displays colored (green (>80 km/h), yellow (40–80 km/h), red (<40 km/h), red/black (very slow, that is stop and go) and gray (no data available)) traffic conditions in real time on major roads and highways by analyzing the GPS-determined locations transmitted to Google by a large number of mobile phone users. Consequently, the speed of the users along a length of road can be calculated and a live traffic map can be produced. Google processes the incoming raw data about mobile phone device locations, but there is no way to tell the form of transportation once the GPS information is received. Thus, this method only roughly indicates the true road condition.

**Figure 5:**
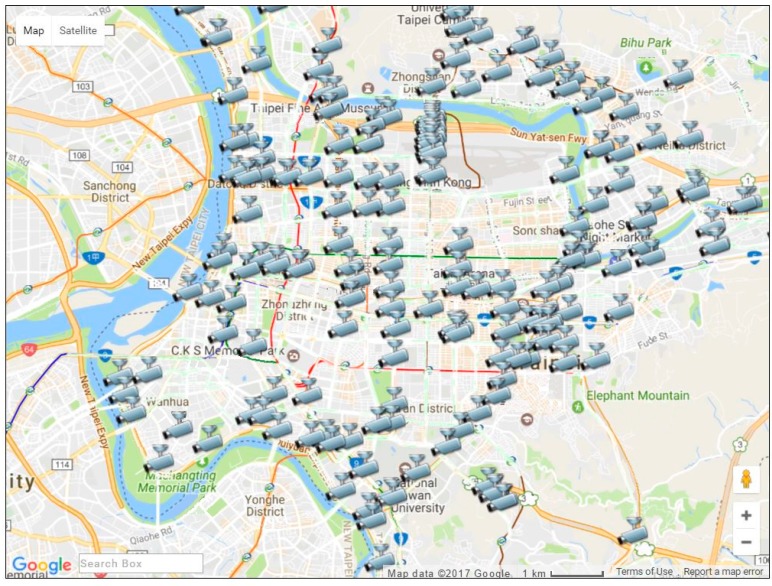
Taipei area traffic CCTV cameras provided by the E-Traffic Center.

The E-Traffic Center of Taipei City provides real-time traffic information. Real-time traffic information includes bus locations, road events, construction sites, subway services, parking lots, and the real-time CCTV camera videos captured from general roads and highways [26]. Figure 5 illustrates locations of real-time traffic CCTV cameras in the Taipei area. By the first quarter of 2014, there were a total of 200 CCTV cameras available in the Taipei area.

**Figure 6:**
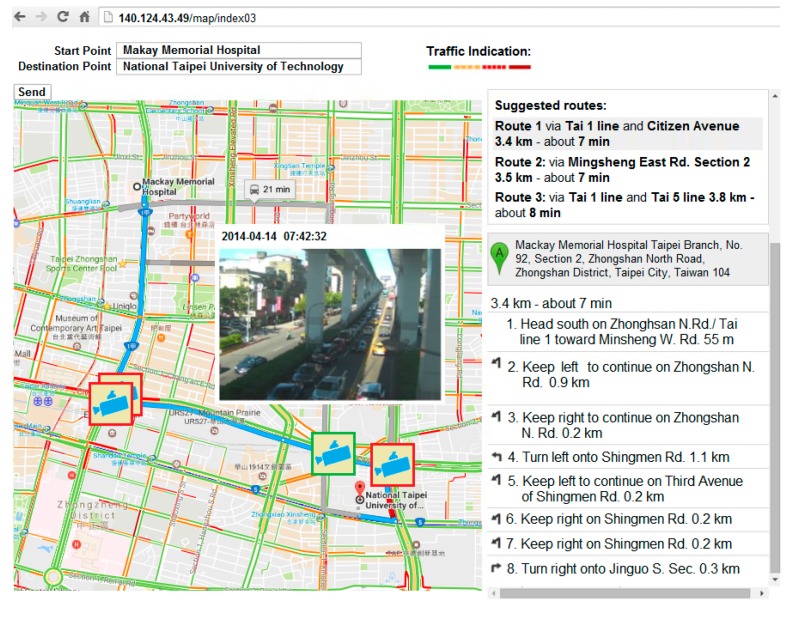
The traffic guiding system. The first route is chosen among the three suggested routes by Google Maps along Civic Boulevard road is congested.

Figure 6 illustrates a snapshot of the TGS. In the figure, the selection of the shortest-time routes is in the right top; selected shortest-time route and a real-time CCTV camera stream among the four available cameras are on the map, and the step-by-step road guiding instruction from a starting point to a destination point are in the right bottom. When the driver chooses one of the routes the TGS will check available CCTV cameras along the chosen route by matching the road names and coordinates of the cameras. In case of matches, camera icons will be displayed on the coordinates of the matched cameras. When the driver touches the icon of a camera, a real-time stream of that particular camera will be displayed on the screen. If the driver chooses another route, then the currently displayed camera icons will disappear and new icons of cameras that are available on that new route will show up on the screen. The TGS displays the current location of the ambulance and records the coordinates in every other second. The recorded coordinates are simultaneously transmitted to and stored in the server-side SQL database.

**Figure 7:**
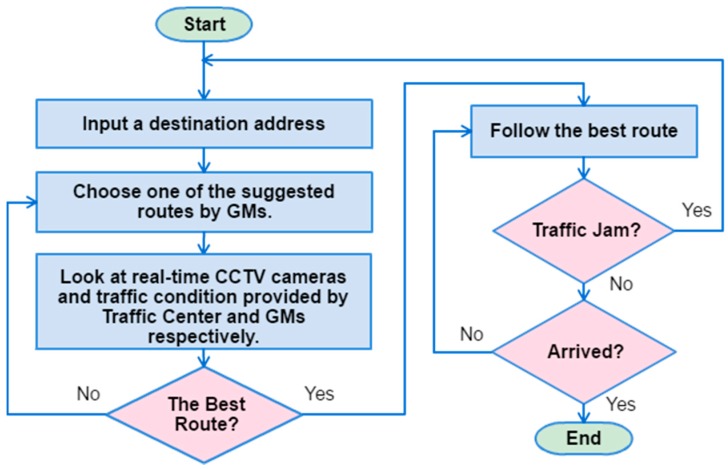
An algorithm to choose the best route.

An algorithm for choosing the best route is depicted in Figure 7. Firstly, the driver inputs a destination address, and waits for the route suggestions. After the suggested routes are displayed, the driver investigates the traffic conditions of all routes. Then, the driver will use his/her intuition based on past experiences to choose the least congested route between those suggested routes. Once the best route is chosen, the driver can drive to the emergency room. In case of traffic congestion on the way to the emergency room, the driver can restart the TGS to find an alternative route to avoid the traffic jam. It is possible that the driver may not choose the best route all the time. For choosing the best route is totally up to the driver’s experience, the available number of CCTV cameras and correctness of traffic condition information.

## 3. Results

### 3.1. Initialization Time

A series of experiments has been conducted in Taipei City. This experiment evaluates an average initialization time that required to start pre-hospital care. The initialization time starts when paramedics attach the wireless sensors to patient’s body at emergency site and ends when the patient’s biosignals and real-time video are displayed on the STI. The time includes connecting to Internet via WiMax/3.5 G, and positioning the ambulance using GPS as well. Notably, the measured SPO_2_, heart rate, and temperature are transmitted with the same bit rate of 8 bits/s. The sampling rate and resolution of the one lead ECG are 500 Hz and 16 bits, resulting in a bit rate of 8 kbits/s. The patient’s video is transmitted at 30 frames/s using quarter-VGA (QVGA) format (320 pixel × 240 pixel) with 24-bit resolution and H.263 compression. The bit rate of the patient’s video is approximately 0.25 Mbits/s.

The experimental setup was an empirical one in which two ambulances were sent by a cooperative hospital during the experiments to match the emergent scenario as closely as possible. Notably, due to the constraint of government regulation, instead of a real patient, a team member played the role of a patient in each ambulance.

The results of a series of field experiments indicate that the average time of 117 s, and 130 s, respectively, are required using WiMax and 3.5 G networks at a driving speed of 60–79 km/h.

### 3.2. Data Rate

This experiment evaluates the maximum achievable transmission data rate (transmission bandwidth) by the WiMax/3.5 G networks under the different ambulance speeds during the field trials. A network traffic analyzer, NetLimiter [27], is used to evaluate data rate. The test results demonstrate that a data rate of at least 0.5 Mbits/s suffices for transmitting the patient’s biosignals and real-time video smoothly. Table 1 illustrates the average transmission rates of WiMax and 3.5 G (HSPA) networks obtained at four driving speeds. The results reveal that the data rate offered by both networks at all driving speeds exceed 0.5 Mbits/s, meeting the requirement for transmitting patients’ biosignals and real-time video. In addition, at driving speeds of 0 km/h, 40–59 km/h, 60–79 km/h, and 80–100 km/h, the actual transmission data rates provided by the WiMax were 2.53, 2.75, 1.62 and 2.22 times greater than those provided by the 3.5 G network, respectively. Notably, the minimum required transmission data rate will increase to 1 Mbits/s for the simultaneous transmission of biosignals and real-time video if the real-time video is transmitted using VGA with H.263 compression. However, the data rate of 0.91 Mbits/s achieved by the 3.5 G network under a driving speed of 80–100 km/h is insufficient for the transmission of both biosignals and videos. Moreover, as shown in Table 1, data rates decrease for both WiMax and 3.5 G networks when the driving speed increases due to the Doppler effect [28].

### 3.3. End-to-End Delay

This experiment measures the average end-to-end delay the hospital server to encounters in receiving the patient’s biosignals and real-time videos sent from the accident site. Table 2 presents the experimental results, which indicate that the end-to-end delay achieved using WiMax at driving speeds of 0 km/h, 40–59 km/h, 60–79 km/h, and 80–100 km/h are 0.1 s, 0.106 s, 0.185 s, and 0.25 s, respectively. However, for the 3.5 G network, the end-to-end delay is 0.25 s, 0.289 s, 0.296 s, and 0.543 s, respectively. Accordingly, the end-to-end delay of the WiMax network is shorter than that of 3.5 G network at each driving speed. Notably, the delay also increases for both networks as the driving speed increases.

### 3.4. Response and Delivery Time

This experiment determines whether the suggested traffic guiding subsystem enables ambulances to avoid congested areas in order to reduce the response and delivery time of the ambulance.

#### 3.4.1. Experimental Setup

**Figure 8:**
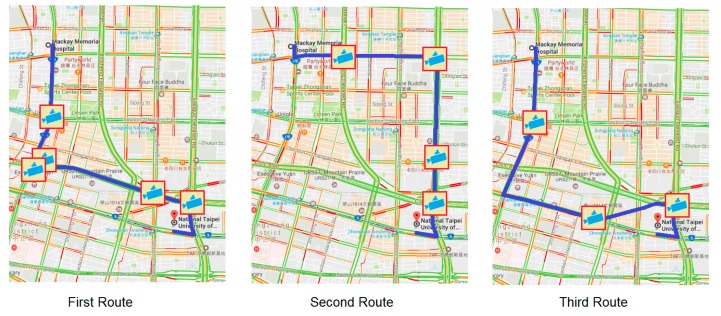
Response routes suggested by Google Maps and available CCTV camera icons.

**Figure 9:**
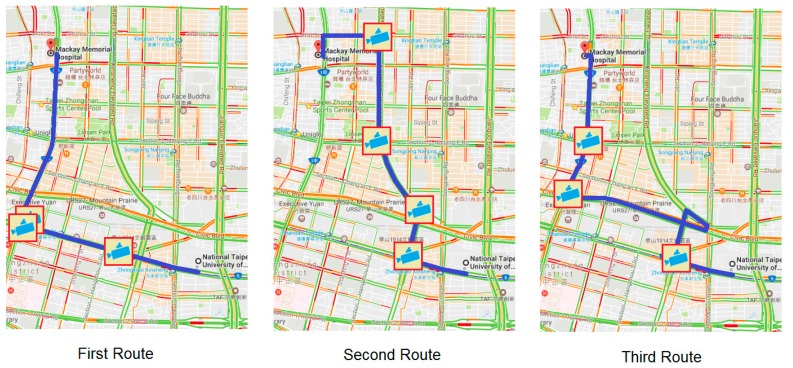
Delivery routes suggested by Google Maps and available CCTV camera icons.

1 Locations: The patient and hospital sites are determined a priori as, respectively, National Taipei University of Technology and Mackay Memorial Hospital.
2 Dates of experiments: 14, 16 and 18 April 2014 (Monday, Wednesday, and Friday). The experimental period consists of three shorter periods: morning rush hour (7:30–8:30 a.m.), noon off-peak hour (12:00 p.m.–1:00 p.m.) and afternoon rush hour (5:30–6:30 p.m.). Traffic conditions are based on the travelling speed: above 60 km/h is specified as clear; 40–60 km/h is specified as semi-congested, and less than 40 km/h is specified as congested.
3 Experimental methods: Two ambulances are involved on different routes (Route 1, Route 2). Route 1 is the first route among the three suggested routes by GMs; Route 2 is a chosen route by the driver after his inspection of the traffic condition. Thus, Route 2 can be the same as Route 1 if the traffic condition of Route 1 is good. Three different routes are suggested by GMs for the response (see Figure 8) and for the delivery (see Figure 9) routes. Both ambulances simultaneously departed from the same location and a stopwatch was used to record their time.

#### 3.4.2. Case I

**Figure 10:**
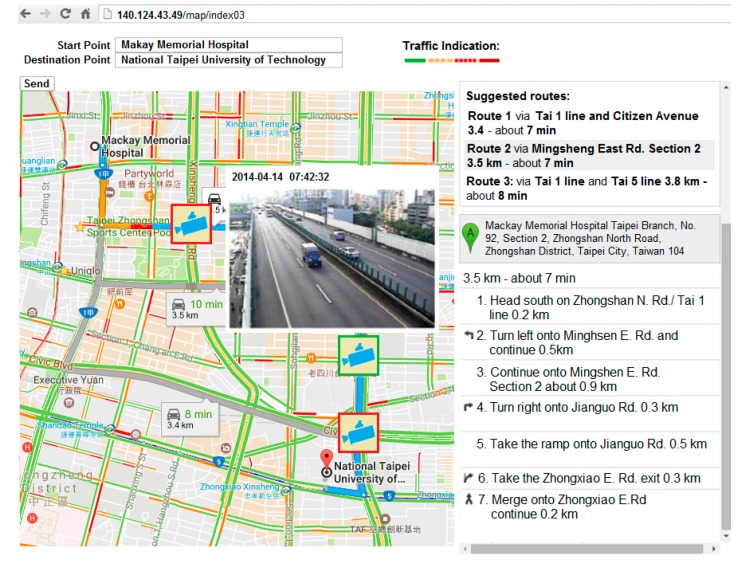
The second suggested route is chosen, along Jianguo elevated road, which is uncongested.

For a response route (from a hospital to an emergency site), during the morning rush hour (7:30–8:30 a.m.). The real-time video revealed congested traffic on Civic Boulevard (see Figure 6) along Route 1. Therefore, the second suggested route was chosen as Route 2 by the driver, because Jianguo elevated road was not congested (see Figure 10) at that time. The measured response time for Route 1 and Route 2 was 932 s, and 625 s, respectively, thus yielding a difference of 307 s. For a delivery route (from an emergency site to a hospital), Route 2 was selected as Route 1, because traffic congestion along Route 1 was light due to the investigation of the camera streams.

This procedure was followed for the experiments on Wednesday and Friday. The three-day average response time was 694 s for Route 1, and 452 s for Route 2 (see Table 3). Average delivery time was 452 s for Route 1, and 343 s for Route 2.

There is an extra time that needs to be added in Route 2, since the driver needs to assess the traffic condition for choosing Route 2. On the other hand, this assessment is not needed for Route 1. The survey result among the drivers indicates that the required extra assessment time is between 3 and 10 s, an average time was 6 s. In Table 3, Route 2 is a sum of the average assessment time (6 s) and the average time between the points (hospital or emergency site).

#### 3.4.3. Case II

In the afternoon off-peak experiment (12:00 p.m.–1:00 p.m.), Jianguo elevated road was selected as Route 1 and Route 2 for both response and delivery routes on all three days. The average response time was 402 s, and average delivery time was 327 s.

#### 3.4.4. Case III

In the afternoon rush-hour experiment (5:30–6:30 p.m.), the real-time video revealed that the traffic on the way to the patient’s location was congested on Jianguo elevated road. Thus, Zhongxiao East Road was selected as Route 2, because this route was uncongested. The average travel time for Route 1 was 756 s, while the average travel time for Route 2 was 500 s, representing a reduction of 256 s. With respect to the delivery route, the average travel time for Route 1 was 588 s, whereas that for Route 2 was 426 s, showing an improvement of 162 s.

### 3.5. Speech Recognition

This experiment evaluates the accuracy of the WSA for Mandarin speakers. Three males and two females aged between 22 and 27, were recruited and asked to speak ten addresses in Chinese. The addresses, listed in Table 4, were in five districts of Taipei city. There were two addresses in each district; one was a residence and the other was a publicly known place, such as a metro station, parks or well-known buildings. Each testee spoke every address ten times, with 100% success in all cases.

## 4. Discussion

To demonstrate the superiority of our proposed UEMS, a comparison with three of the most similar EMS systems [8,11,12] was made. Kyriacou et al. [8] proposed a multi-purpose healthcare telemedicine system. Even though a large number of biosignals and still images of the patient are transmitted through the GSM mobile network, the average transmission time for an image, ranging from 18 to 26 s through the GSM link, is unacceptable for timely emergency medical service. On the other hand, the longest video transfer time in our system were only 23 s and 9 s through 3.5 G and WiMax networks, respectively, under the driving speed of 80–100 km/h. Notably, instead of still images, video is transmitted in our system. Alesanco et al. [12] investigated the issue of wireless ECG transmission for real-time cardiac tele-monitoring. A robust transmission technique was developed to deal with the retransmission of the erroneous packets, and evaluated the transmission and monitoring process from a clinical point of view. A fairly limited variety of biosignals were considered in this work, and the major pitfall relies on computer simulation in the feasibility validation, where a two-state Markov model is adopted to simulate the wireless channel. This makes a drastic difference from our field-proved approach previously presented in our work. Thelen et al. [11] implemented an integrated telemedicine system prototype for real-time patient monitoring by employing commercially available off-the-shelf devices and software. The signal delays of ECG and SaO_2_ waveforms between patient monitor to tele-EMS physician display were measured via simulated 2 G/3 G networks, and a median of 4.9 s end-to-end signal delayed was reported, which exceeded the maximum acceptable delay of 4 s suggested [12]. In the absence of field testing, simulation alone cannot reflect truthfully the real-word scenario, such as the Doppler effect due to the ambulance driving at a high speed, signal degradation caused by multipath fading, and QoS fluctuation due to bandwidth competition among mobile users and cell handoff. According to our experience, the delay performance reported will worsen even more in field validation. This rather unacceptable delay leaves much to be desired in a highly time-critical life-saving circumstance. In contrast, our field-test results revealed the longest delay time for simultaneously transmitting all biosignals and patient video at 80–100 km/h driving speed via 3.5 G and WiMax networks were 4.634 and 1.563 s, respectively [29].

Electromagnetic radiation hazards must be concerned with the wireless biosensors because they are closely attached to the human body. According to ICNIRP (International Commission on Non-Ionizing Radiation Protection) guidelines, suggested maximum electromagnetic field exposure levels for general public and occupational places are 61 V/m and 137 V/m [30], respectively. By calculation, our Zigbee module’s electric field at 1 cm distance from the antenna is around 30 V/m, which is 2.03 and 4.6 times lower than the ICNIRP limits for general public and occupational places, respectively. In addition, according to a manufacturer’s user guide [31] of the XBee 2mW Zigbee module, the module can be attached to human body if the antenna gain is less than 13.8 dBi. Our Zigbee module’s antenna gain is around 1.5 dBi, thus, we consider our wireless system is not hazardous to the human body.

During the trial-experiments, performances of the telemedicine devices, i.e., the initialization time, the data rate, and the end-to-end delay are evaluated for both WiMax and 3.5 G networks. As a result, both WiMax and 3.5 G networks are deemed to be acceptable for the emergency telemedicine services owing to their sufficient data rate at different driving speeds (except the scenario of 80–100 km/h for 3.5 G), and a very short end-to-end delay.

Response time and delivery time are decreased by using the TGS. In the morning rush hours, average response and delivery time of Route 2 were 34.87%, and 9.97% shorter as compared to Route 1, respectively. Similarly, in the evening rush hours, average response and delivery time of Route 2 were 33.86% and 27.55% shorter as compared to Route 1, respectively.

A door-to-needle time is another crucial part of the emergency medical services. It is a time between the time of patient’s arrival at the hospital (defined as the “door time”) and the time of start of the treatment (defined as the “needle time”). Generally, noted by experienced emergency room doctors, 720 s (12 min) on average is typically required to diagnose the patient’s condition based on the received patient’s biosignals and video. Therefore, in case of the 1200 s (20 min) delivery time, including the initialization time, the proposed system provides 363 s with the WiMax network and 350 s with the 3.5 G network for doctors to conduct emergency medical preparation before the ambulance arrives at the hospital. As a result, the door-to-needle time is significantly decreased and the patient’s treatment can be started immediately.

## 5. Conclusions and Future Work

This work developed a novel UEMS system based on traffic information, wireless biosignals, wireless communication, Google Maps, and speech recognition technologies. A series of field trials was carried out using WiMax and 3.5 G networks under various driving speeds to verify the effectiveness of the proposed system. The experimental results reveal that the WiMax network outperformed the 3.5 G network in terms of transmission data rate and normalized completion time for the hospital to receive the patient’s biosignals and real-time videos. Additionally, although the first routes that were recommended by GMs were shorter than the others, they were sometimes congested during the morning and evening rush hours. The routes that were identified by the TGS were clear of traffic, improving rescue time by 34.87% and 31.21%, respectively, over those achieved using the routes that were suggested by GMs.

The results show that the proposed UEMS system enables the patients to be sent delivered to hospitals in the shortest possible time, significantly increasing the effectiveness of first aid. Extensive interviews with emergency room doctors concerning the applicability of UEMS system yielded positive responses. Therefore, the proposed system is useful for reducing preparation time for treating the patients in emergency cases, thus, greatly enhancing the quality of emergency medical services.

Our proposed system can be implemented in practice without any difficulties from the view point of information and communication technologies (ICT). However, a major difficulty possibly encountered is the accuracy of choosing the best route by using traffic guiding subsystem due to insufficient CCTV cameras in some areas and incorrect traffic information provided by GMs.

In the future, the data transmitted, namely, videos, images, and biosignals of the patient, all belong to highly sensitive private health information which will be Health Insurance Portability and Accountability Act (HIPAA) compliant and encrypted using AES (Advanced Encryption Standard) to prevent any leakage during the process of wireless transmission and unauthorized usage. Ethical guidelines shall be followed for the protection of human subjects to obtain the delicate balance between risks and benefits of human subjects.

To reduce the time required for data coding and decoding, in the current prototypic system, all the acquired biosignals and real-time videos of the patient are transmitted to the server in raw data format since the wireless network offers sufficient data rate (bandwidth). However, as the demand for more biosignals and higher video resolution grows, the bandwidth available will be unenviably insufficient. In the future, a more sophisticated scheme to extract critical features of the biosignals in the client site before transmitting to the remote server to reduce transmission loading will be devised.

## Figures and Tables

**Table 1:** Transmission data rates of WiMAX and 3.5 G networks at various driving speeds.

Ambulance Driving Speed (km/h)	WiMAX (Mbits/s)	3.5 G (Mbits/s)	Ratio Rate_WiMAX_/Rate_3.5G_
0	4.98	1.97	2.52
40–59	4.71	1.71	2.75
60–79	2.71	1.67	1.62
80–100	2.02	0.91	2.21

**Table 2:** Average end-to-end delay required for hospital to receive patient’s data.

Ambulance Driving Speed (km/h)	WiMAX (s)	3.5 G (s)	Difference Time_3.5G_–Time_WiMax _(s)
0	0.100	0.254	0.154
40–59	0.106	0.292	0.186
60–79	0.185	0.299	0.114
80–100	0.248	0.549	0.301

**Table 3:** Comparison of response and delivery time along Routes 1 and 2.

Execution Period	Response Time			Delivery Time		
	Route 1	Route 2	Difference	Route 1	Route 2	Difference
7:30–8:30 a.m.	694 s	458 s	236 s	452 s	349 s	91 s
12:00–1:00 p.m.	402 s	408 s	–6 s	327 s	333 s	–6 s
5:30–6:30 p.m.	756 s	506 s	250 s	588 s	432 s	144 s

**Table 4:** Addresses selected from five districts in Taipei City.

No.	Name (Address) in English
1	Taipei Fine Arts Museum (No. 181, Section 3, Zhongshan N. Road, Zhongshan Dist., Taipei City)
2	No. 181, Section 3, Zhongshan N. Road, Zhongshan Dist., Taipei City
3	National Taiwan University Hospital (No. 7, Chung Shan S. Road (Zhongshan S. Road, Zhongzheng Dist., Taipei City)
4	No. 7, Chung Shan S. Road (Zhongshan S. Rd, Zhongzheng Dist., Taipei City)
5	Taipei Zoo (No. 30, Section 2, Xinguang Road, Wenshan Dist., Taipei City)
6	No. 30, Section 2, Xinguang Road, Wenshan Dist., Taipei City
7	National Palace Museum (No. 221, Section 2, Zhishan Road, Shilin Dist., Taipei City)
8	No. 221, Section 2, Zhishan Road, Shilin Dist., Taipei City
9	Taipei 101 (No. 7, Section 5, Xinyi Road, Xinyi Dist., Taipei City)
10	No. 7, Section 5, Xinyi Road, Xinyi Dist., Taipei City
